# Therapeutic Actions of Hepatocyte Extracellular Vesicles in a Murine Model of Diet-Induced Steatohepatitis with Fibrosis

**DOI:** 10.3390/biomedicines13020274

**Published:** 2025-01-23

**Authors:** Xinlei Li, Ruju Chen, Sherri Kemper, Zhaohui Xu, David R. Brigstock

**Affiliations:** 1 Center for Clinical and Translational Research, The Abigail Wexner Research Institute at Nationwide Children’s Hospital, Columbus, OH 43205, USA; xinlei.li@nationwidechildrens.org (X.L.); rujuc9889@gmail.com (R.C.); sherri.kemper@nationwidechildrens.org (S.K.); 2 Department of Infectious Diseases, St. Jude Children’s Research Hospital, Memphis, TN 38105, USA; zhaohui.xu@stjude.org; 3 Department of Surgery, Wexner Medical Center, The Ohio State University, Columbus, OH 43212, USA

**Keywords:** MASLD, MASH, extracellular vesicle, exosome, hepatocyte, liver, fibrosis, therapy

## Abstract

Introduction: Metabolic dysfunction-associated steatohepatitis (MASH) is a leading cause of liver failure globally and is characterized by hepatic steatosis and inflammation, which may progress to fibrosis, the severity of which is highly predictive of patient demise and death. In view of the lack of treatment options for MASH, we investigated the therapeutic properties of extracellular vesicles (EVs) from normal human hepatocytes, which we have previously been shown to alleviate toxin-mediated hepatic fibrosis in mice. Methods: C57BI/6J mice were fed a choline-deficient amino acid-defined high (60%) fat (CDAA-HF) diet for up to 12 weeks while receiving i.p. administration of EVs purified from cultured human HepG2 hepatocytes. Results: CDAA-HF diet consumption resulted in severe hepatic steatosis, increased frequency of CD45+ lymphocytes and F4/80+ macrophages, robust production of aortic smooth muscle actin (ACTA2), and deposition of interstitial collagen, as well as altered serum levels of ALT, AST, cholesterol, triglycerides, alkaline phosphatase, unconjugated bilirubin, and total protein, thus recapitulating typical MASH phenotypes. EVs administered preventively or therapeutically resulted in the restoration of serum marker levels, reduced hepatic inflammation and attenuation of collagen deposition, ACTA2 production, and expression of fibrosis-associated genes. HepG2 EVs contained 205 miRs and, among the 30 most abundant miRs, seven (miRs-423-5p, -483-5p, -191-5p, -148a-3p, -423-3p, -92a-3p, -122-5p) are predicted to directly target fibrosis-related genes (*collagen*s, *ACTA2*, *MMP*s, and *TIMP*s). Conclusions: Hepatocyte EVs are therapeutic in a mouse model of diet-induced steatohepatitis with fibrosis. Further studies of hepatocyte EVs or their cargo components as novel therapeutics for MASH in humans are warranted, including treatment of fibrotic stages, which are associated with clinical demise and are predictive of patient death.

## 1. Introduction

Metabolic dysfunction-associated steatotic liver disease (MASLD) is a chronic disease of the liver that is characterized by hepatic fat accumulation without concurrent liver disease and the presence of at least one cardiometabolic risk factor [1]. An advanced form of the diseased, termed metabolic dysfunction-associated steatohepatitis (MASH), is characterized by hepatic lobular ballooning and inflammation with or without fibrosis of varying severity, and it is increasingly recognized as an emerging and highly burdensome public health issue [2,3]. In the USA, about 25% of the population is affected by MASLD [3], while MASH with moderate-to-advanced liver fibrosis affects 6–8 million people [4], with that number expected to increase. MASH can progress to liver cirrhosis and/or hepatocellular carcinoma (HCC), which are the leading causes of liver-related mortality. In early 2024, the first drug to treat MASH was approved by the U.S. Food and Drug Administration (FDA) [5]. However, other therapeutic treatment options are still urgently needed.

Liver fibrosis is the only histologic variable that independently predicts liver-related morbidity or mortality and all-cause death in MASLD patients [6]. Although there are various animal models available for MASH pathogenesis research and drug discovery, very few of these models recapitulate liver fibrosis pathogenesis. Western high-fat diet with fructose and cholesterol—for example, the Gubra Amylin NASH (GAN) diet (40% fat, 22% fructose, 10% sucrose, and 2% cholesterol)—feeding in C57BI/6 mice for up to 44 weeks results in enhanced body and liver weights, metabolic disorder, liver macro-steatosis, and inflammation, but hepatic fibrosis is mild and develops slowly, only becoming evident after about 10 months; this is a serious disadvantage of this and similar models for testing drugs for MASH, especially those with potential anti-fibrotic actions [7]. By contrast, methionine- and choline-deficient (MCD) or choline-deficient amino acid defined (CDAA) diet feeding in conjunction with high fat resulted in steatosis and inflammation, as well as, respectively, moderate or progressive liver fibrosis by week 8 [8], and these models are thus advantageous for accelerated progress in MASH drug discovery.

Extracellular vesicles (EVs) are membranous nanovesicles which contain a complex molecular payload (miRs, mRNAs, lipids, and proteins) that reflects the transcriptional and translational activity of their producer cells [9]. Delivery of EVs to recipient cells may result in functional alterations in the cells, depending on the molecular information received. In the liver, both parenchymal (hepatocytes) and non-parenchymal cells (e.g., hepatic stellate cells (HSC), macrophages, and sinusoid endothelial cells) release EVs under physiological or pathological conditions [10]. In diet-induced MASH models, EV release is increased according to MASH disease severity [11]. Recent studies have implicated EVs in promoting lipid accumulation in hepatocytes through mechanisms that involve EV cargo components such as miR-122, which promotes cholesterol and fatty acid synthesis [12]; HMGB1, which is transported in EVs from the intestine to liver, where it promotes steatosis [13]; miR-199a-5p or aldo-keto-reductase Ib7, which are enriched in ER-stressed adipose tissue during MASH and delivered in EVs to hepatocytes, where they promote hepatic steatosis and inflammation [14,15]; and miR-106b-5p from macrophages, which inhibits mitofusion expression, leading to hepatocyte mitochondrial dysfunction, impaired energetics, and enhanced lipid accumulation [16]. Other studies have shown that lipid-laden hepatocytes release EVs at a higher rate than normal hepatocytes, and that these EVs drive inflammatory responses by promoting hepatic macrophage recruitment and production of the pro-inflammatory M1 phenotype, which has been attributed, in part, to EV miR-122-5p and miR-192-5p, the latter of which targets the Rictor/Akt/FoxO1 pathway [17,18,19,20,21]. Finally, EVs released from lipid-laden hepatocytes also promote HSC activation [22] and induce angiogenesis in endothelial cells [23]. Collectively these studies strongly implicate EVs in driving multiple features of MASH.

While EVs from MASH-associated hepatocytes drive many aberrant processes and pathogenic features of the disease, EVs from normal non-injured hepatocytes, on the other hand, play a role in hepatic homeostasis and have protective and therapeutic properties. For example, we showed that EVs from normal non-injured mouse or human hepatocytes efficiently reverse injury, inflammation, and fibrosis in the livers of mice that were chronically exposed to carbon tetrachloride (CCl_4_) [24]. This finding raises the intriguing possibility that EVs from non-injured hepatocytes are similarly therapeutic in models of MASH for which there are very limited treatments options, despite the profound medical and financial burden of the disease. To address this question, EVs were purified from human hepatocytes and administered prophylactically or therapeutically via i.p. injection in mice that were concurrently fed a CDAA-HF diet for up to 12 weeks. Analysis of serum and liver tissues revealed that hepatocyte EVs reduced MASH-associated serological and hepatic components relating to liver injury, steatosis, altered lipid metabolism, inflammation, and fibrosis, the latter of which was attributed at least partly to the predicted action of EV miRs that target fibrosis-related mRNAs.

## 2. Materials and Methods

### 2.1. MASH Disease Model

Animal protocols were approved by the Institutional Animal Care and Use Committee of The Research Institute at Nationwide Children’s Hospital (Columbus, OH, USA). Male C57BL/6J mice (8 weeks old; n = 5 per group) purchased from the Jackson Laboratory were given either choline-deficient amino-acid defined (CDAA) diets with high (60%) fat (CDAA-HF (Catalog #A06071302, Research Diets Inc., New Brunswick, NJ, USA) for 4, 8, or 12 weeks, as we previously described [25], or normal chow food (2020X, Inotiv, West Lafayette, IN, USA). Some mice received EVs (4 × 10^8^ particles or 2 × 10^9^ particles) via i.p injection once (Monday), twice (Monday and Friday), or three times (Monday, Wednesday, and Friday) each week for the entire duration of CDAA-HF feeding (preventative dosing strategy), while other mice received EVs (1 × 10^9^ particles) 3 times per week (Monday, Wednesday, and Friday) for the last 6 weeks of an 8-week CDAA-HF diet (therapeutic dosing strategy). At the termination of the experiment, liver tissues were harvested after ketamaine/xylazine anesthesia. In some cases, blood was collected via cardiac puncture, and the livers were perfused through the left heart ventricle sequentially with PBS and 4% paraformaldehyde (PFA). One lobe of the liver was tied off with suture material after PBS perfusion and perfused livers were collected and weighed. Liver lobes were then used for paraffin embedding or were snap-frozen in liquid nitrogen for subsequent RNA extraction for real time quantitative polymerase chain reaction (RT-qPCR).

### 2.2. Histology and Immunofluorescence

Perfused mouse livers were fixed and embedded in paraffin. Paraffined sections of 5 μm thickness were cut and stained with hematoxylin and eosin (H and E) for histology, or with 0.1% Sirius Red (Sigma-Aldrich, St. Louis, MO, USA) for detection of collagen. Some slides underwent immunostaining with antibodies to F4/80 (Ca# 70076S,1:250, Cell Signaling, Danvers, MA, USA), CD45 (Cat# 70257S, 1:500, CST), ACTA2 (Cat# A700-082-T, 1:500; Thermo Fisher Scientific, Waltham, MA, USA), followed by Alexa Fluor 488 goat-anti mouse IgG, Alexa Fluor 568 goat-anti mouse IgG, Alexa Fluor 568 goat-anti rabbit IgG, or Alexa Fluor 488 goat-anti-rabbit IgG (all at 1:500; Thermo Fisher Scientific) for 1 h at room temperature. The slides were counterstained with 4′,6-diamidino-2-phenylindole (DAPI, 1:1000; Thermo Fisher Scientific) and mounted in glycerin jelly. The slides were examined by confocal microscopy, and positive signals were quantified using ImageJ (1.53k, National Institutes of Health, Bethesda, MD, USA).

### 2.3. Liver Function Tests

Blood was allowed to clot prior to centrifugation at 5000× *g* for 10 min to collect serum, which was and tested for alanine aminotransferase (ALT), aspartate aminotransferase (AST), cholesterol, triglyceride, total protein, alkaline phosphatase, unconjugated bilirubin, and conjugated bilirubin levels (IDEXX Laboratories, Westbrook, ME, USA).

### 2.4. Isolation and Characterization of EVs from HepG2 Cells

Human hepatocyte HepG2 cells (HB-8065, American Type Culture Collection, Manassas, VA, USA) were cultured with Dulbecco’s Modified Essential Medium (DMEM) supplemented with 10% fetal bovine serum and antibiotic-antimycotic. Cells were then switched to serum-free medium for 48 h before the culture supernatants were collected. Using our established methodology [26], EVs were purified by sequential differential centrifugation (300× *g* for 10 min, 2000× *g* for 20 min, 10,000× *g* for 30 min, and two steps of 100,000× *g* for 70 min), resuspended in DPBS, and characterized nanosight tracking analysis (NTA) using a Nanosight 300 instrument (Malvern Instruments, Westborough, MA, USA). Two videos were recorded for each sample, and NTA 3.2 Dev Build 3.2.16 software was used to estimate particle size and frequency. The recordings were performed at room temperature using a camera gain of 13, a shutter speed of 4.13 ms, and a detection threshold of 4. EV protein concentration was determined using a Pierce bicinchoninic acid (BCA) assay (Thermo Fisher Scientific). A total of 30 μg of EV or cell lysates were mixed with RIPA lysis buffer (Thermo Fisher Scientific) and subjected to sodium dodecyl sulfate–polyacrylamide gel electrophoresis on 10% gels and Western blot, with each sample being run in 3 separate lanes. Blots were cut and individual lanes were incubated with antibodies to CD63 (Cat# CBL553, Sigma Millipore, St. Louis, MO, USA), ALIX (Cat# MA1-83977, Thermo Fisher), CD9 (Cat# ab92726, Abcam, Cambridge, UK), Tsg101 (Cat# MA1-23296, Thermo Fisher), cellular marker Calnexin (Cat# NB100-1965SS, NOVUS, Centennial, CO, USA), or β-actin (Cat# MA1-140 Invitrogen, Waltham, MA, USA). Secondary antibodies were IRDye 800CW goat anti-mouse (Cat# 9a26-32210, LI-COR Biotechnology, Lincoln, NE, USA) or anti-rabbit (Cat# 926-32211, LI-COR). Blots were developed with a ChemiDoc MP imaging system (Bio-Rad, Hercules, CA, USA).

### 2.5. RNA Extraction and RT-qPCR

Total RNA from liver tissues, HepG2 cells, or HepG2 EVs was extracted using miRNeasy mini kits (Qiagen, Germantown, MD, USA). To measure fibrotic transcript expression in liver tissues, the RNA was subjected to one-step RT-qPCR using CFX Connect Real-Time PCR System and one-step iTaq Universal SYBR Green Supermix (Bio-Rad). Primers are shown in Table 1. For miR expression detection in HepG2 EVs or cells, RNA was first reverse transcribed to complementary DNA (cDNA) via miRCURY LNA RT kit (Cat# 339340, Qiagen), and the cDNA was then used for real-time PCR using proprietary miRCURY LNA miR PCR primers (Cat# 339306, Qiagen). Each reaction was run in duplicate, and samples were normalized to *GAPDH* or *UniSp6*.

### 2.6. EV miR Sequencing

Three different batches of HepG2 EVs were collected and used for RNA extraction with miRNeasy mini kits. Approximately 200 ng of RNA was subjected to Multiplex Small RNA Library preparation using NEBNext^®^ Small RNA Library Prep Set according to the manufacturer’s instructions. The library was then run on an Illumina MiniSeq platform. Quality control of the sequencing data was performed using miRTrace version 1.0.1 software, and the miR counts were normalized and subjected to target searching and functional prediction by miRWalk, an online open-source platform (http://mirwalk.umm.uni-heidelberg.de/ accessed on 14 November 2024) that generates predicted or validated miR-binding target sites by searching complete transcript sequences (which includes 3′UTR, 5′UTR, and coding sequence) with the random-forest-based approach software, TarpmiR [27].

### 2.7. Statistical Analysis

Experiments were carried out at least twice in duplicate or triplicate and data were expressed as mean ± SEM. Immunofluorescence images were quantified using ImageJ. RT-qPCR and imaging data were analyzed by student’s *t*-test. A *p*-value < 0.05 was considered statistically significant.

## 3. Results

### 3.1. Establishment of MASLD Mouse Model

**Figure 1:**
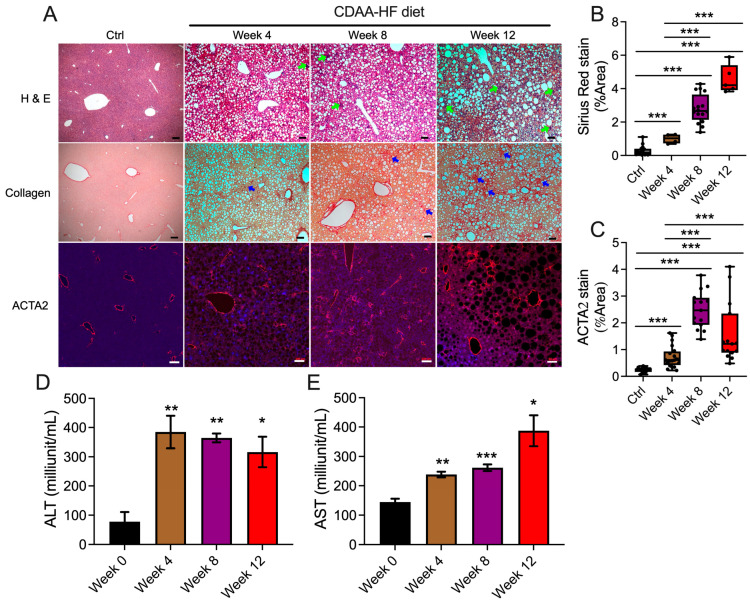
Histological, fibrotic, and functional changes in livers of mice fed a CDAA-HF diet. C57Bl/6J mice were fed a CDAA-HF diet for 4, 8, or 12 weeks after which they were sacrificed. The same age mice fed with chow food for 8 weeks were used as control. (**A**) Livers were collected, sectioned, and stained with H&E, Sirius Red, or anti-ACTA2, the latter being developed with fluorescent secondary antibody. Green arrows indicate inflammatory infiltration aggregates; blue arrows show interstitial collagen deposition. Scale bar = 100 μm (Ctrl, week 4 or 8) or 50 μm (week 12). Blue staining is DAPI. (**B**) Collagen deposition was quantified via ImageJ analysis of Sirius Red staining. (**C**) ACTA2 intensity was quantified via ImageJ analysis of ACTA2 immunofluorescence. Liver function tests were performed on serum for (**D**) ALT or (**E**) AST. * *p* < 0.05, ** *p* < 0.01, *** *p* < 0.005.

C57BI/6J male mice were fed with a CDAA-HF diet or chow food for 4, 8, or 12 weeks. Consistent with a previous report [8], 4 weeks of feeding with a CDAA-HF diet resulted in hepatocyte lipid droplet accumulation, enhanced immune cell infiltration, collagen deposition, and ACTA2 expression in the interstitial area (Figure 1A–C). Steatosis, collagen deposition, and ACTA2 expression increased over time during the 12 weeks of CDAA-HF feeding (Figure 1A). ACTA2 staining was principally associated with vessels in control animals, becoming more abundant and spreading interstitially to areas of weaker DAPI staining as hepatocyte lipid accumulated in CDAA-HF-fed mice. Consistent with the histological changes in the liver, serum ALT and AST levels were significantly elevated after 4 weeks of CDAA-HF feeding and remained consistently high for up to 12 weeks (Figure 1D,E). None of these changes were evident in control mice that were fed a normal chow diet (Figure 1A–C). CDAA-HF-fed mice also demonstrated alterations in serum liver function and metabolic components, as described below.

### 3.2. HepG2 EVs Ameliorate Fibrosis in MASLD Mice

**Figure 2:**
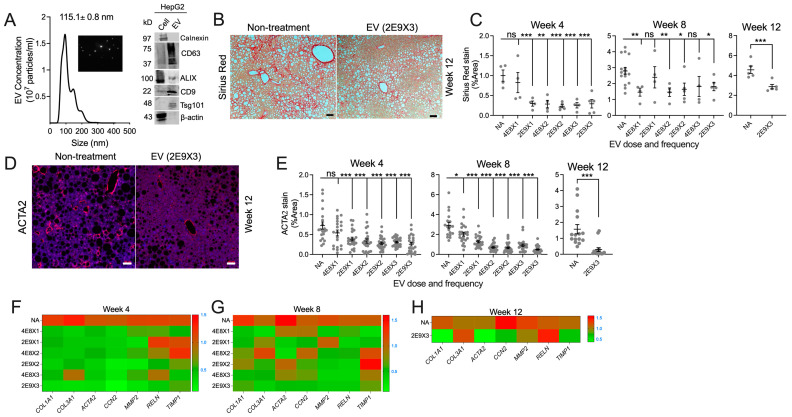
HepG2 EVs reduce collagen deposition, ACTA2 production, and lipid metabolism gene expression. (**A**) HepG2 EV characterization via NTA (left; the inset shows a typical video frame from the analysis) and Western blot (right). Original raw data are in Supplemental Movie S1 and Supplemental Figure S1. (**B**) Collagen deposition detected via Sirius Red staining in liver sections from CDAA-HF-fed mice with no treatment, or which received HepG2 EV treatment of 2 × 10^9^ particles three times a week for 12 weeks (scale bar = 100 μm). (**C**) ImageJ quantification of collagen deposition in mice receiving EV doses of 4 × 10^8^ particles or 2 × 10^9^ particles, once (“X1”), twice (“X2”), or three times (“X3”) per week for 4, 8, or 12 weeks. (**D**) ACTA2 detection by immunofluorescence assay of livers from (**B**). Blue staining is DAPI. (**E**) ImageJ quantification of ACTA2 intensity in mice from (**D**). Frozen liver tissue from mice maintained on a CDAA-HF diet for (**F**) 4, (**G**) 8, or (**H**) 12 weeks were subjected to RNA extraction, reverse transcription, and quantitative detection of extracellular matrix gene *COL1A1*, *COL3A1*, *ACTA2*, *CCN2*, *MMP2*, *RELN*, and *TIMP1* via real-time PCR. The y-axis indicates EV dose, with the frequency of administration per week denoted as “X1” (once), “X2” (twice), or “X3” (three times). * *p* < 0.05, ** *p* < 0.01, *** *p* < 0.005. ns, non-significant.

EVs isolated from 48-hour serum-free conditioned medium from HepG2 cells had a mean diameter of 115 nm and were positive for CD63, ALIX, CD9, and Tsg101 (EV markers) but were negative for calnexin or β-actin, demonstrating a lack of appreciable contamination with cellular/cytosolic material (Figure 2A). Typical visual features of the EVs, as recorded during NTA, are shown in a captured video frame (Figure 2A inset) and in the video recording (Supplemental Movie S1). The original Western blot raw data are in Supplemental Figure S1.

HepG2 EVs were then injected one, two, or three times a week at two different doses during the entire time (4, 8, 12 weeks) for which C57BI/6J mice received a CDAA-HF diet (preventative model). Typical Sirius red staining is presented in the representative panels in Figure 2B, which shows that the high levels of collagen in mice receiving a CDAA-HF diet for 12 weeks were substantially reduced when the mice were concurrently administered 2 × 10^9^ EVs three times a week. The more complete analysis is shown in Figs 2C, in which data from each time point (4, 8, 12 weeks) were quantified by ImageJ and expressed as a function of EV dose (4 × 10^8^ or 2 × 10^9^ particles) and frequency (1, 2, or 3 i.p. injections per week). These data convincingly show that most EV dosing regimens were highly effective in reducing hepatic collagen. Whereas EVs at the 4 × 10^8^ particle dose once per week at week 4 or the particle doses of 2 × 10^9^ or 4 × 10^8^ at, respectively, once or three times per week at Week 8 were not effective in reducing collagen (Figure 2C), these data points appear to be spurious outliers; all other EVs doses were highly significant in their ability to suppress collagen deposition. Comparable data for ACTA2, a surrogate of HSC activation and fibrogenesis, revealed a consistent therapeutic attenuation of ACTA2 expression across all dosing parameters against a background of increasing DAPI staining which likely reflected improved hepatocyte viability, reduced steatosis, and parenchymal restoration (Figure 2D,E). These outcomes were reinforced via transcriptional analysis of expression of genes related to extracellular matrix and fibrosis (*COL1A1*, *COL3A1*, *ACTA2*, *CCN2*, *MMP2*, *RELN*, and *TIMP1*), which were suppressed by the EVs, sometimes in a complex manner that varied according to time, dose, and frequency of dosing (Figure 2F–H). Overall, these results showed that HepG2 EVs were preventative for fibrosis-related outcomes in the livers of mice on a CDAA-HF diet, including HSC activation, fibrosis, and ECM gene expression.

### 3.3. HepG2 EVs Ameliorate Immune Cell Infiltration

**Figure 3:**
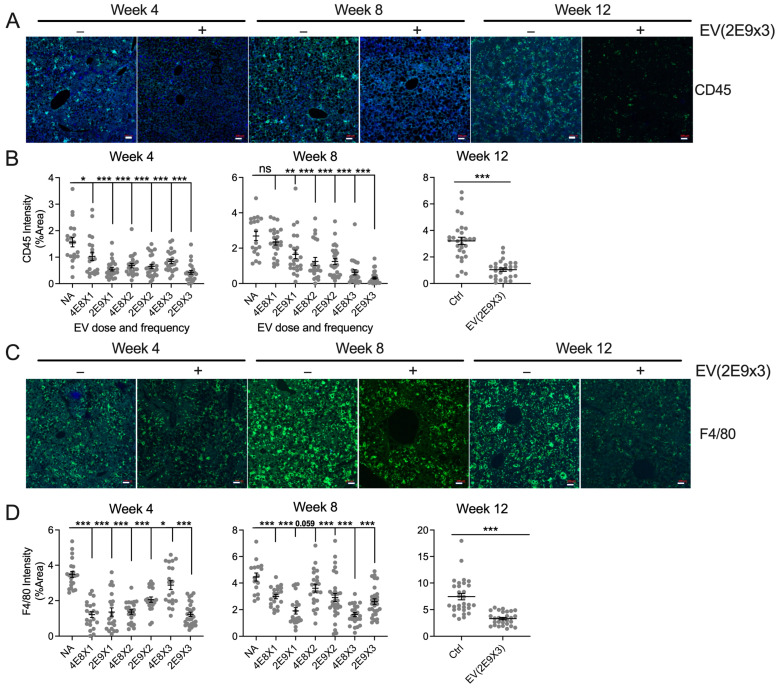
HepG2 EVs ameliorate liver inflammation. (**A**) Immunofluorescent detection of CD45 in liver sections from mice with no treatment or which were treated with HepG2 EVs at 2 × 10^9^ particles three times per week for 4, 8, or 12 weeks. (**B**) ImageJ quantification of CD45 intensity in mice receiving EV doses of 4 × 10^8^ or 2 × 10^9^ particles for 4, 8, or 12 weeks; the frequency of EV administration per week is shown as “X1” (once), “X2” (twice), or “X3” (three times). (**C**) Immunofluorescent detection of F4/80 in liver sections from mice with no treatment or which were treated with HepG2 EVs at 2 × 10^9^ particles three times per week for 4, 8, or 12 weeks. (**D**) ImageJ quantification of F4/80 intensity in mice receiving EV doses of 4 × 10^8^ or 2 × 10^9^ particles for 4, 8, or 12 weeks; the frequency of EV administration per week is shown as “X1” (once), “X2” (twice), or “X3” (three times). Scale bar = 100 μm for 4, 8 weeks, and 50 μm for 12 weeks. * *p* < 0.05, ** *p* < 0.01, *** *p* < 0.005. ns, non-significant.

As assessed via immunofluorescent staining, there were increased levels of CD45 (lymphocyte marker) and F4/80 (macrophage marker) over the 12-week duration of CDAA-HF feeding (Figure 3A–D). Virtually every EV dosing regimen at all three time points in the preventative model resulted in a significant attenuation of CD45 and F4/80 signals, showing that CDAA-HF-induced inflammation and immune cell infiltration were effectively suppressed by the EVs.

### 3.4. HepG2 EVs Improve Liver Function

**Figure 4:**
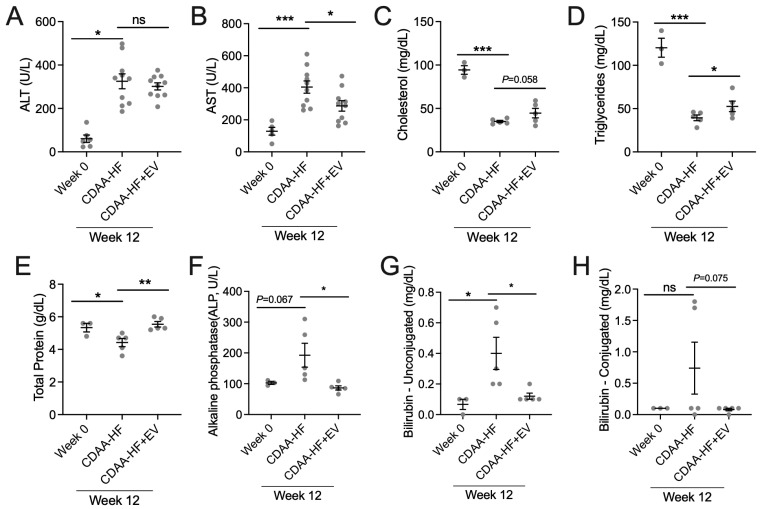
HepG2 EVs improve liver function serum markers. Serum levels of (**A**) ALT, (**B**) AST, (**C**) cholesterol, (**D**) triglycerides (**E**) total protein, (**F**) alkaline phosphatase, (**G**) unconjugated bilirubin, and (**H**) conjugated bilirubin in CDAA-HF-fed mice treated with or without HepG2 EVs (2 × 10^9^ particles three times per week) for 12 weeks. * *p* < 0.05, ** *p* < 0.01, *** *p* < 0.005. ns, non-significant.

The CDAA-HF diet-induced increase in serum ALT at 12 weeks (Figure 1B) was slightly but non-significantly reversed by HepG2 EVs in the preventative model (Figure 4A), while the increased AST levels (Figure 1C) were significantly suppressed after EV administration (Figure 4B). In mice fed a CDAA-HF diet for 12 weeks, HepG2 EVs administered in the preventative model caused the suppressed level of serum cholesterol (Figure 4C) and triglycerides (Figure 4D) to be partially reversed, although the improvement was not significant in the case of cholesterol. Mice receiving a CDAA-HF diet for 12 weeks also had alterations in serum total protein (significant increase), alkaline phosphatase (upwardly trending), or unconjugated bilirubin (significant increase), and all such changes were significantly reversed by HepG2 EVs in the preventive model (Figure 4E–G). There were no significant changes in serum conjugated bilirubin in mice receiving a 12-week CDAA-HF diet with or without HepG2 EV administration (Figure 4H).

### 3.5. HepG2 EVs Are Therapeutic After Onset of MASLD

**Figure 5:**
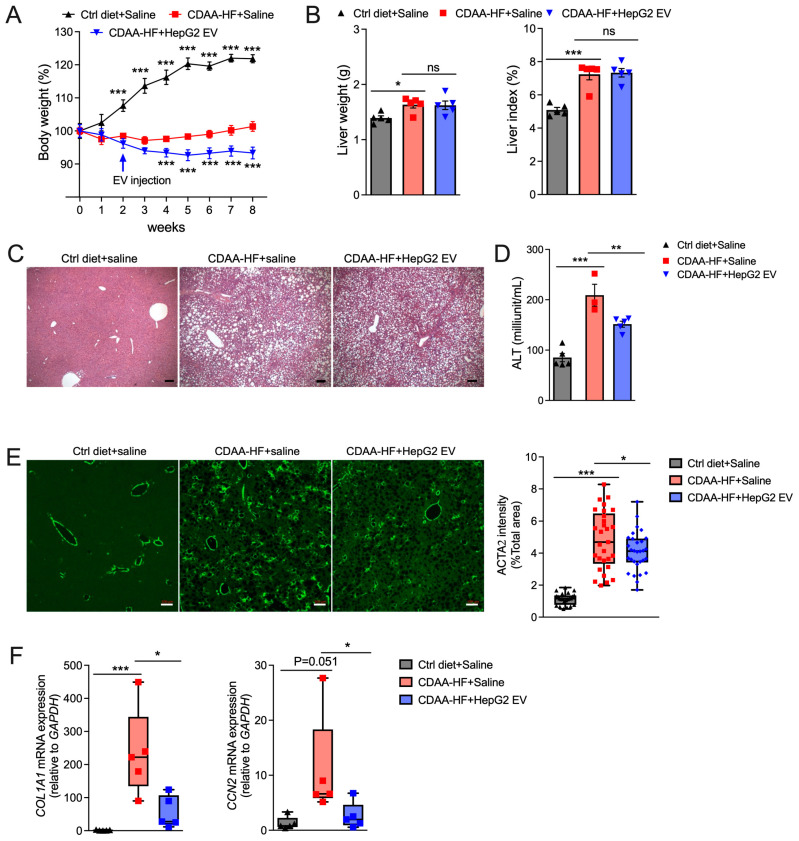
Therapeutic effects of HepG2 EV treatment in MASH mice. Mice were given a CDAA-HF diet or chow food for 8 weeks and HepG2 EVs or saline were administered from the end of the second week for 6 weeks. After sacrifice, livers were perfused, weighed, fixed, and processed for histology and RNA analysis, and blood was collected for serum analysis. (**A**) Weekly body weights normalized to body weight on first day of, but prior to, initiation of CDAA-HF feeding. (**B**) Liver index (liver weight/body weight). (**C**) H&E staining (scale bar = 50 μm). (**D**) Serum ALT. (**E**) Representative ACTA2 confocal immunostaining (scale bar = 100 μm); the panel to the right represents quantification of at least 3 fields from each mouse using ImageJ. (**F**) RT-qPCR for *COL1A1* and *CCN2*. * *p* < 0.05, ** *p* < 0.01, *** *p* < 0.005. ns, non-significant.

To test the therapeutic ability of EVs after onset of injury, mice were administered EVs (1 × 10^9^, 3 times a week) for the last 6 weeks of an 8-week CDAA-HF diet. EVs efficiently reduced the diet-induced body weight of mice (Figure 5A), even though the enlarged livers or the liver index were not significantly altered by the EVs (Figure 5B). Compared to the non-treated group, HepG2 EV-treated livers showed less large-droplet (macrovesicular) steatosis (Figure 5C). Consistently, elevated serum ALT level reduced significantly after HepG2 EV treatment (Figure 5D), which also caused a dramatic attenuation of ACTA2 protein production (Figure 5E) and reduced expression of liver fibrosis-associated genes including *COL1A1* and *CCN2* (Figure 5F).

### 3.6. HepG2 EV miR Profiling

**Figure 6:**
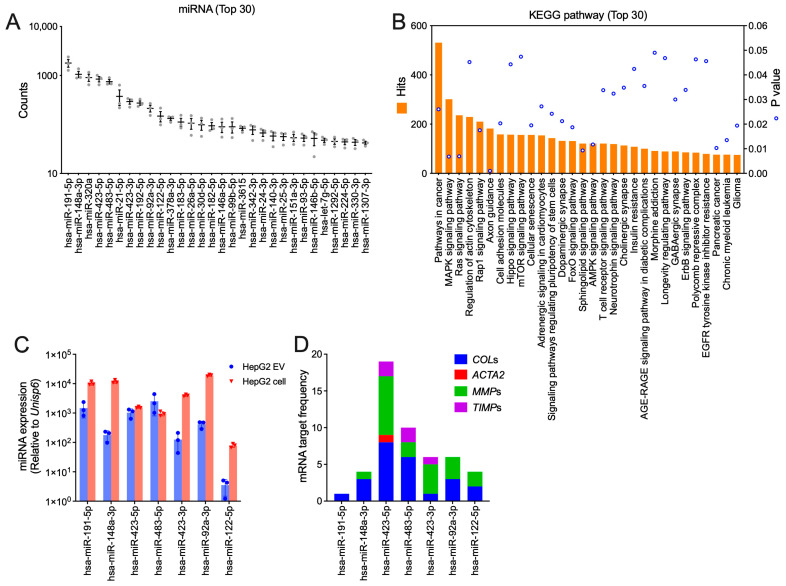
HepG2 EV miR profile and function prediction. Total HepG2 EV RNA was isolated and miR content and identity was determined by Illumina small RNA sequencing. (**A**) The most abundant 30 miRs out of 205 total are shown. (**B**) The 10 most abundant miRs from (**A**) were selected to search for potential mRNA targets on miRWalk, and a total of 10,636 predicted target genes were used for KEGG enrichment analysis. The top 30 KEGG pathways are shown. (**C**) RT-qPCR of selected miRs in HepG2 EVs or cells that are predicted to target (**D**) genes encoding fibrosis-associated RNAs (*collagen*s, *ACTA2*, *MMP*s, and *TIMP*s).

miRs are important bioactive components in EVs payloads but can also serve as regulators, biomarkers, or therapeutic targets in liver diseases [28]. HepG2 EV RNA was isolated and subjected to small RNA sequencing for miR profiling. A total of 205 miRs were identified (Supplemental Table S1), with miR-191-5p, miR-148a-3p, miR-320a, miR-423-5p, and miR-483-5p ranked as the top five most abundant miRs (Figure 6A). Next, the 30 most abundant miRs were searched on miRWalk for their potential targets; 10,636 predicted genes were retrieved, which were predicted by KEGG analysis to be involved in cancer; signaling via MAPK, Ras, or Rap1; or regulation of the actin cytoskeleton (Figure 6B). The presence of seven abundant miRs (miR-191-5p, miR-148a-3p, miR-423-5p, miR-483-5p, miR-423-3p, miR-92a-3p, and miR-122-5p) in HepG2 EVs, as well as in the HepG2 producer cells, was confirmed by RT-qPCR miR (Figure 6C), and they are of interest because they are predicted to have mRNA targets that are fibrosis-related (*collagen*s, *ACTA2*, *MMP*s, and *TIMP*s). miR-423-5p was predicted to have the largest target repertoire; as many as 19 gene targets were identified, which included 8 collagen genes, 8 *MMP*s, 2 *TIMP*s, and *ACTA2*. miR-483-5p was predicted to target 10 such genes, including 6 collagen genes, 2 *MMP*s, and 2 *TIMP*s (Figure 6D). Finally, several other miRs in HepG2 EVs, including miR-191-5p, miR-148a-3p, miR-423-3p, miR-92a-3p, and miR-122-5p, were also predicted to target between one and seven fibrosis-related gene targets (Figure 6D). The full list of miR and their predicted transcript binding sites is shown in Supplemental Table S2.

## 4. Discussion

CDAA-HF diet feeding represents one of the most rapid and robust approaches to induce liver fibrosis in models of MASLD in C57BI/6 mice [8,10]. In this study, progressive collagen deposition and ACTA2 production was seen as early as 4 weeks after initiation of feeding, becoming progressively more advanced after 8 or 12 weeks. These features allowed us to determine anti-fibrotic actions of hepatocyte EVs in this MASLD mouse model using preventative or therapeutic approaches. In the preventative approach, hepatocyte EVs were administered upon initiation of CDAA-HF diet feeding over 4-, 8-, or 12-week periods and resulted in attenuation of diet-induced collagen deposition, ACTA2 expression, pro-fibrotic gene expression, infiltration of lymphocytes, and macrophages, as well as improvements in serum AST or triglyceride levels. In the therapeutic approach, EVs were administered two weeks after the initiation of an 8-week CDAA-HF feeding regimen. As compared to their non-treated counterparts, mice receiving EV treatment demonstrated less lipid accumulation, reduced liver injury, and dramatic suppression of ACTA2 induction and pro-fibrotic gene expression. Collectively, the data indicate that hepatocyte EVs not only prevent but, importantly, can also reverse liver fibrosis in MASLD mice.

We are not aware of prior reports in which hepatocyte EVs have been shown to be therapeutic in MASLD models, but EVs from mesenchymal stem cells have been studied in this regard. Anti-steatotic, anti-inflammatory, and anti-oxidant actions and regulation of associated gene expression have been demonstrated in mice and rats that were treated with mesenchymal stem cell EVs and exposed to diets such as methionine–choline-deficient, high-fat, or high-fat, high-cholesterol [29,30,31,32,33,34,35,36], or in LPS-treated melanocortin-4 receptor-deficient (Mc4r-KO) mice maintained on a Western diet (a rapid MASH model) [30]. EV components, including several miRs (e.g., miR 24-3p, 96-5p, 223-3p, 627-5p) [31,32,33,35] and one protein (CAMKK1) [36], were implicated in mediating some of the anti-steatotic or anti-inflammatory actions in lipid-laden hepatocytes. However, a drawback in these studies is that animal models were used in which the fibrotic component is slow to develop and/or mild, and scant information was reported regarding MSC EV anti-fibrotic actions. While certain anti-fibrotic actions were noted, such as suppressed collagen expression or reduced Sirius red staining [30,35], underlying mechanisms were not addressed. The results of this current investigation show that several therapeutic properties of hepatocyte EVs are similar to those of stem cell EVs, but the robust fibrotic response in our mouse model allowed us to demonstrate strong anti-fibrotic aspects of EVs, which are of particular importance given the clinical importance of MASH fibrosis for patient outcomes.

HepG2 EVs contained several abundant miRs including miR-191-5p, miR-148a-3p, miR-320a, miR-423-5p, and miR-483-5p, which may contribute to their ability to prevent or reverse the various MASLD-related pathologies. Given the importance of fibrosis as a prognostic indicator in MASH, we used miRWalk to screen for EV miRs that target fibrosis-related genes such as *collagen*s, *ACTA2*, *MMP*s, and *TIMP*s based on the predicted presence of miR-binding sites in the 3′-UTR or 5′-UTR, which, upon binding by the specific miR, results in post-transcriptional cleavage or repression of the respective target gene. MiR-423-5p and miR-483-5p were particularly notable because they are predicted to target, respectively, 19 and 10 components in these gene families, with miR-191-5p, miR-148a-3p, miR-423-3p, miR-92a-3p, and miR-122-5p also predicting to have potential fibrosis-related gene targets. Consistent with these predictions, HepG2 EVs were previously shown to contain a high level of miR-423-5p, which exerted an anti-fibrotic effect in HSC, which was reduced in the EVs after cellular stress with ethanol or lauric acid [37]. Anti-fibrotic actions of miR-483-5p have been similarly reported in recent studies from this and other laboratories. For example, we reported that miR-483-5p is more highly expressed in serum EVs from normal mice versus fibrotic mice or from healthy human subjects versus hepatitis B patients with F3/F4 fibrosis [38]. We showed that a miR-483-5p mimic suppressed expression of *ACTA2*, *COL1A1*, and *CCN2/CTGF* in activated HSC and proposed that the presence of miR-483-5p in serum EVs from healthy individuals contributed to the anti-fibrotic actions of these EVs in mouse CCl_4_ fibrosis models and HSC activation in vitro [38]. In other studies, miR-483-5p and its complementary strand miR-483-3p were identified from a human embryonic liver [39], and a significantly decreased expression of miR-483 was observed in fibrotic livers in rat fed with choline-deficient methionine supplemented diet for 4 weeks [40]. A follow-up study demonstrated that miR-483 overexpression in CCl_4_-injured mouse livers inhibited liver fibrosis, with both miR-483-5p and miR-483-3p having anti-fibrotic activities that, respectively, targeted *PDGF-β* and *TIMP2* [41]. Finally, retrospective clinical analysis found that serum miR-148a-3p levels were negatively correlated with TGF-β or FIB4 liver fibrosis scores in HCC patients with hepatitis C virus infection, suggesting that high miR-148a-3p levels are associated with prolonged survival [42], but it remains to be seen whether the inhibitory effect of miR-148a-3p on TGF-β, a key cytokine that plays central role in the development of liver fibrosis, was direct or indirect. A potential limitation or complication of our findings is that components with inhibitory or stimulatory effects on fibrosis are predicted to be regulated by the same miR, such as miR-148a-3p, which targets *COL10A1*, which is pro-fibrotic, but also *MMP15* and *MMP16*, which may be anti-fibrotic. The biological outcome in such instances will be the focus of our future studies, which will involve the up- or down-regulation of these key miRs in an effort to establish their individual and collective functional roles, as well as their specific molecular targets and roles in fibrosis.

Prior studies have shown that EVs from normal non-injured hepatocytes can stimulate hepatocyte proliferation and repopulation [43], as well as suppressing fibrogenesis in HSC in vitro, inhibiting CCl_4_-mediated fibrosis in vivo, and reversing the suppression of proliferation of, or altered gene expression in, hepatocytes exposed to CCl_4_ or ethanol [24,44]. On the other hand, EVs produced by hepatocytes that are stressed, injured, infected, or tumorigenic have quite different properties, which are pro-pathogenic and promote disease processes. EVs from HBV-, HCV-, or HEV-infected hepatocytes move viruses between hepatocytes [45,46,47], promote T cell production, drive HSC activation [48,49], and stimulate macrophage activation and immune function, the latter of which also occurs after exposure of the cells to alcohol [50,51,52]. EVs from CCl_4_-treated hepatocytes drive a positive feedback loop between HSC and T cells involving a TLR3-IL-17A axis that enhances liver fibrosis [53], while EVs from free fatty acid- or lysolecithin-treated hepatocytes stimulate chemotaxis and pro-inflammatory molecule production by macrophages [17,18], endothelial cell tube formation and angiogenesis [23], and HSC activation [22,54]. The ability of hepatocytes to produce either therapeutic or pathogenic EVs, depending on the environmental conditions, suggest that the balance between these various EV populations is an important factor in disease progression and further suggests that hepatocyte EVs may play a protective role in dampening the impact of injury on hepatocyte function and protecting the liver from downstream deleterious signaling (inflammation, fibrosis). Future studies will address this important putative homeostatic property, as well as defining the inflection point at which the hepatocyte EV population becomes pro-pathogenic after various types of liver injury.

In conclusion, the results of this study show that liver injury, altered liver function, steatosis, inflammation, fibrosis, and fibrosis-related gene expression are ameliorated by hepatocyte EVs when administered preventatively or therapeutically in a mouse MASH-like model. In view of the association between liver fibrosis severity and the clinical demise of the MASH patient, the anti-fibrotic actions of hepatocyte EVs identified in this study may have particular importance for clinical utility and may be attributable to high levels of EV miRs that are predicted to target genes central to fibrosis development.

## Figures and Tables

**Table 1:** Primers used for RT-qPCR.

Gene ID	Accession No.	Primer		Length (bp)
		Fwd Seq (5′-3′)	Rev Seq (5′-3′)	
*CCN2*	NM_010217	CACTCTGCCAGTGGAGTTCA	AAGATGTCATTGTCCCCAGG	111
*COL1A1*	NM_007742	GCCCGAACCCCAAGGAAAAGAAGC	CTGGGAGGCCTCGGTGGACATTAG	148
*ACTA2*	NM_007392	GGCTCTGGGCTCTGTAAGG	CTCTTGCTCTGGGCTTCATC	148
*COL3A1*	NM_009930	GCCCACAGCCTTCTACACCT	GCCAGGGTCACCATTTCTC	110
*GAPDH*	NM_002046	TGCACCACCAACTGCTTAGC	GGCATGGACTGTGGTCATGAG	87
*TIMP1*	NM_001044384	CCTATAGTGCTGGCTGTGGG	GCAAAGTGACGGCTCTGGTA	136
*MMP2*	NM_008610	GCAGCTGTACAGACACTGGT	ACAGCTGTTGTAGGAGGTGC	182
*RELN*	MMU24703	TTACTCGCACCTTGCTGAAAT	CAGTTGCTGGTAGGAGTCAAAG	73

## Data Availability

The original contributions presented in this study are included in the article/Supplementary Material. Further inquiries can be directed to the corresponding author. The small RNA-seq datasets generated for this study can be found in the GEO accession GSE286556.

## Supplementary Materials

The following supporting information can be downloaded at https://www.mdpi.com/article/10.3390/biomedicines13020274/s1: Table S1. Identity and abundance of miRs in HepG2 EVs; Table S2. Predicted binding sites in fibrosis-related targets of selected EV miRs; Movie S1. Video recording of HepG2 EV nanosight tracking analysis; Figure S1. Original Western blot raw data.
