# Supplementary figures and images for: Therapeutic Actions of Hepatocyte Extracellular Vesicles in a Murine Model of Diet-Induced Steatohepatitis with Fibrosis
Source: Biomedicines. 2025 Jan 23;13(2):274. doi: 10.3390/biomedicines13020274 (PMC11852249; doi:10.3390/biomedicines13020274)

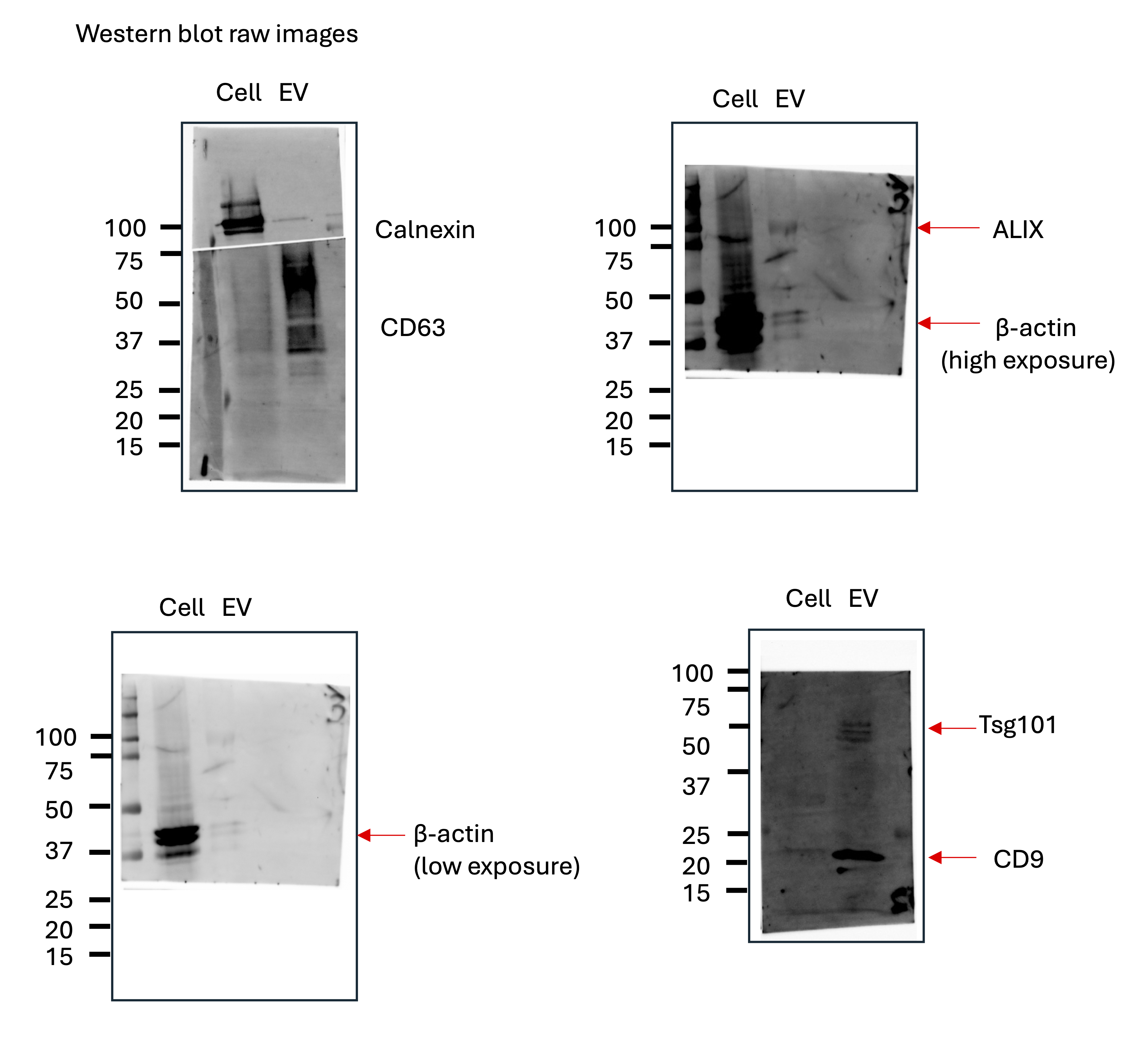

Supplement: Supplementary file 1 [file biomedicines-13-00274-s001.zip › Supplemental Figure S1.tiff]
